# A Randomized Trial Comparing Concurrent versus Sequential Radiation and Endocrine Therapy in Early-Stage, Hormone-Responsive Breast Cancer

**DOI:** 10.3390/curroncol31080338

**Published:** 2024-08-07

**Authors:** Sharon F. McGee, Mark Clemons, Gregory Pond, Jean-Michel Caudrelier, Michelle Liu, Mashari Jemaan Alzahrani, Terry L. Ng, Arif A. Awan, Sandeep Sehdev, John Hilton, Marie-France Savard, Lesley Fallowfield, Vikaash Kumar, Orit Freedman, Lisa Vandermeer, Brian Hutton, Jean-Marc Bourque

**Affiliations:** 1 Department of Medicine, Division of Medical Oncology, The Ottawa Hospital, University of Ottawa, Ottawa, ON K1H 8L6, Canada; mclemons@toh.ca (M.C.); jcaudrelier@toh.ca (J.-M.C.); mashari_220@hotmail.com (M.J.A.); teng@toh.ca (T.L.N.); aawan@ohri.ca (A.A.A.); jfhilton@toh.ca (J.H.); msavard@toh.ca (M.-F.S.); jean-marc.bourque.med@ssss.gouv.qc.ca (J.-M.B.); 2 Cancer Therapeutics Program, Ottawa Hospital Research Institute, Ottawa, ON K1H 8L6, Canada; miliu@ohri.ca (M.L.); lvandermeer@ohri.ca (L.V.); bhutton@ohri.ca (B.H.); 3 Department of Oncology, McMaster University, Hamilton, ON L8S 4L8, Canada; gpond@mcmaster.ca; 4 SHORE-C, Brighton & Sussex Medical School, University of Sussex, Brighton BN1 9RH, UK; l.j.fallowfield@sussex.ac.uk; 5 Markham Stouffville Hospital, Markham, ON L3P 7P3, Canada; vikaash.kumar@uhn.ca; 6 Lakeridge Health, Oshawa, ON L1G 8A2, Canada; ofreedman@lh.ca; 7 Centre Hospitalier de l’Universite de Montréal (CHUM), Montreal, QC H2X 0A9, Canada

**Keywords:** breast cancer, endocrine therapy, radiotherapy, treatment sequence, toxicity, quality of life

## Abstract

Concerns exist regarding increased toxicities, including endocrine therapy toxicity, with concurrent radiation and endocrine therapy in early breast cancer (EBC). We present a pragmatic, randomized trial comparing concurrent versus sequential endocrine and radiotherapy in hormone-responsive EBC. In this multicenter trial, patients were randomized to receive adjuvant endocrine therapy concurrent with, or sequential to, radiotherapy. The primary outcome was change in endocrine therapy toxicity from baseline to 3 months post radiotherapy using the Functional Assessment of Cancer Therapy–Endocrine Symptom (FACT-ES) score. From September 2019 to January 2021, 133 patients were randomized to concurrent endocrine and radiotherapy, and 127 to sequential treatment. Most patients were post-menopausal (72.7%, 189/260) with stage 1 disease (65.8%, 171/260). Tamoxifen was the endocrine therapy of choice for 69.6% (181/260) of patients, and an aromatase inhibitor for the remainder. The median total radiation dose and fractions were 40.1 Gray (range 26–50) and 15 fractions (range 5–25), respectively. For the primary outcome of change in endocrine therapy toxicity per FACT-ES scores from baseline to 3 months post radiotherapy, no significant difference was found between the groups (median [range] = −4.9 (−82, 38.8) for concurrent and −5.1 (−42, 40) for sequential, *p* = 0.87). This is the first trial to investigate the impact of concurrent versus sequential adjuvant endocrine and radiotherapy on endocrine therapy-related toxicities. The findings provide further support to allow the optimal timing of radiation and endocrine therapy to be tailored for the individual patient.

## 1. Introduction

The optimal time to start adjuvant endocrine therapy relative to radiotherapy in patients with hormone-responsive early breast cancer (EBC) is unknown, with patients beginning endocrine therapy either before, during, or after radiotherapy. A systematic review of concurrent versus sequential adjuvant radiation and endocrine therapy conducted by our group showed no significant difference in efficacy or toxicity with concurrent versus sequential radiation and endocrine therapy [1]. However, this was based on a paucity of high-quality data, with just one fully completed and published prospective, randomized trial, which investigated the sequence of adjuvant radiation and aromatase inhibition with letrozole [2,3].

Recent guidelines stated that radiation may be delivered safely during endocrine therapy [4]. However, in a survey of physicians, we found that most respondents (57%, 36/65) administered endocrine therapy sequentially after radiotherapy. Thirty-two percent (21/65) reported concerns regarding concurrent radiation and endocrine therapy. The primary reasons for concern cited were potential increased side effects with endocrine therapy (62%, 13/21), reduced efficacy of radiotherapy (19%, 4/21), reduced endocrine therapy compliance (14%, 3/21), and increased radiation toxicity (5%, 1/21) [5]. In an institutional consensus statement on the optimal timing of adjuvant radiation and endocrine therapy in EBC, Cecchini et al. also reported physicians’ “anecdotal experience” that concurrent radiation and endocrine therapy were more symptomatically difficult for patients to tolerate, with increased endocrine therapy toxicities interfering with treatment. This concern contributed to their recommendation for sequential treatment [6]. 

The lack of evidence guiding these clinical practices highlighted the need for definitive, randomized data, which respondents to our survey supported. Furthermore, concerns regarding the impact of treatment sequence on endocrine therapy toxicity and compliance represent an important clinical question for both patients and healthcare providers [7,8], which has not been reported in the literature to date. Indeed, the REaCT-RETT trial was noted as being of particular interest in a recent review, where authors felt the data would provide a more complete understanding of additional toxicities that may result from concurrent radiation and endocrine therapy [9].

Here, we present the results of REaCT-RETT, a pragmatic, prospective, randomized clinical trial comparing concurrent versus sequential adjuvant endocrine and radiotherapy in hormone-responsive EBC. The primary outcome was the difference in endocrine therapy toxicity between concurrent and sequential treatment groups. Secondary endpoints included quality of life (QOL), radiation toxicity, and patient-reported compliance. The underlying hypothesis was that there would be no significant difference between the treatment groups for the primary and secondary endpoints.

## 2. Materials and Methods

### 2.1. Study Design

The study was a multicenter, pragmatic, prospective, randomized clinical trial, with patients accrued across 3 sites in Ontario, Canada. Patients were randomized to receive either concurrent radiation and endocrine therapy or sequential treatment with radiation followed by endocrine therapy. ClinicalTrials.gov registration: NCT0394856.

### 2.2. Patients

Patients were approached to participate by either their radiation or medical oncologist during routine clinical visits. Eligible patients had newly diagnosed early stage (I–III), hormone-responsive breast cancer and were planned to receive both adjuvant radiation and endocrine therapy. It was felt to be important to include stage III patients, as due to the higher risk of systemic relapse, they are likely to benefit most from timely initiation of adjuvant systemic endocrine therapy concurrent with radiation. Finally, patients were required to be at least 18 years of age and able to provide verbal consent. The trial utilized the integrated consent model, incorporating verbal consent [10,11].

### 2.3. Randomization

Patients were randomized 1:1 using a permuted block design of variable block sizes of 4 and 6. Allocation was performed by the physician in the clinic, or by a clinical research associate, using a web-based application developed by The Ottawa Methods Centre. Assignment to the treatment groups was stratified by center and whether patients had received chemotherapy.

### 2.4. Procedures

There was no study-mandated change in either the choice of endocrine therapy (i.e., tamoxifen and/or AI and/or luteinizing hormone-releasing hormone analogues) or locoregional radiotherapy prescribed. Concurrent endocrine therapy was defined as the commencement of endocrine therapy 1 to 4 weeks before commencement of radiotherapy, while sequential endocrine therapy commenced 1 to 4 weeks after the last fraction of radiotherapy. Outcome data were collected at baseline (maximum 2 weeks before or 1 week after starting either radiation or endocrine therapy, whichever was first), end of radiation (maximum 3 weeks after the day the patient received the final fraction of radiation), 3 months (±2 weeks), 6 months (±2 weeks), and 12 months (±2 weeks) post the final fraction of radiotherapy.

### 2.5. Questionnaires and Assessments

Endocrine toxicity and QOL assessments were performed using the FACT system and EQ-5D-5L. The FACT–Endocrine Symptoms (ES) questionnaire (version 4) includes the Endocrine Symptom Subscale (ESS), which assesses endocrine therapy toxicities across 4 symptom categories: vasomotor, neuropsychological, gastrointestinal, and gynecological [12]. FACT-B (version 4) measures general QOL associated with cancer (FACT-General [G]), with additional breast cancer-specific questions (Breast Cancer Subscale [BCS]) [13]. FACT-G has 5 subscales assessing physical well-being (PWB), social well-being (SWB), emotional well-being (EMW), functional well-being (FWB), and relationship with the physician [14]. The EQ-5D-5L measures QOL in 5 domains including mobility, self-care, usual activities, pain/discomfort, and anxiety/depression, with an additional question on overall health [15].

Radiotherapy toxicity was assessed using the validated Radiation Toxicity Assessment Tool [16], where the Common Terminology Criteria for Adverse Events (CTCAE) (version 5) were used to grade the following toxicities: rash, induration, pain, telangiectasia, breast swelling, fat necrosis, chronic mastitis, dyspnea, and pneumonitis. Global breast cosmesis was rated according to the European Organization for Research and Treatment of Cancer (EORTC) system [17]. 

Physician and patient questionnaires assessed whether patients initiated and remained on endocrine therapy, and patient preference for the timing of therapies.

### 2.6. Outcomes

The primary outcome was the change in endocrine therapy toxicity from baseline to 3 months post radiation using the validated total FACT-ES score (a summation of FACT-G and FACT-ES scores) [18,19]. The 3-month timepoint was selected as it has been identified as the period of greatest potential endocrine therapy toxicity [18].

Secondary outcomes included the change from baseline to 3 months post radiation in the trial outcome index (TOI: a summation of FACT-PWB, FWB, and BCS scores) [18,19], a validated, breast cancer-specific QOL assessment. Change from baseline to other study timepoints (end of radiation, 6, and 12 months post radiation) for total FACT-ES, FACT-TOI, and EQ-5D-5L QOL scores was also assessed.

### 2.7. Sample Size

The primary outcome was the difference in median total FACT-ES scores from baseline to the 3 months post radiation, with the primary analysis based on an analysis of covariance (ANCOVA), adjusted for stratification factors. Assuming a total of 176 patients were accrued (88 per group), then an ANCOVA would have 80% power (α = 0.05) to detect effect sizes as small as 0.25, regardless of the level of correlation with covariates. Assuming a 10% drop-out rate, the target for accrual was set at 218 patients. This sample size was felt to be sufficient to detect any minimally important differences (MIDs), which have previously been validated for total FACT-ES and TOI scores as a 7.5 and 5-point absolute difference, respectively [13,20,21].

### 2.8. Statistical Analysis

Descriptive statistics were used to summarize patient, disease, and treatment characteristics, as well as outcomes, by allocated intervention arm. The primary analysis was based on an ANCOVA adjusted for stratification factors. Exploratory comparisons between groups were examined using a Chi-squared test for dichotomous events, and a Student’s *t*-test or non-parametric methods for continuous measures as appropriate. All analyses were supplemented with 95% confidence intervals where relevant. Tests were two-sided and a *p*-value of 0.05 or less was considered statistically significant. Analyses were performed using all available data and details of any missing data are described. Supportive analyses for the primary outcome were performed using multiple imputation via MCMC methods, and assuming all patients with missing data had no changes in scores between baseline and month 3.

## 3. Results

### 3.1. Study Conduct

**Figure 1:**
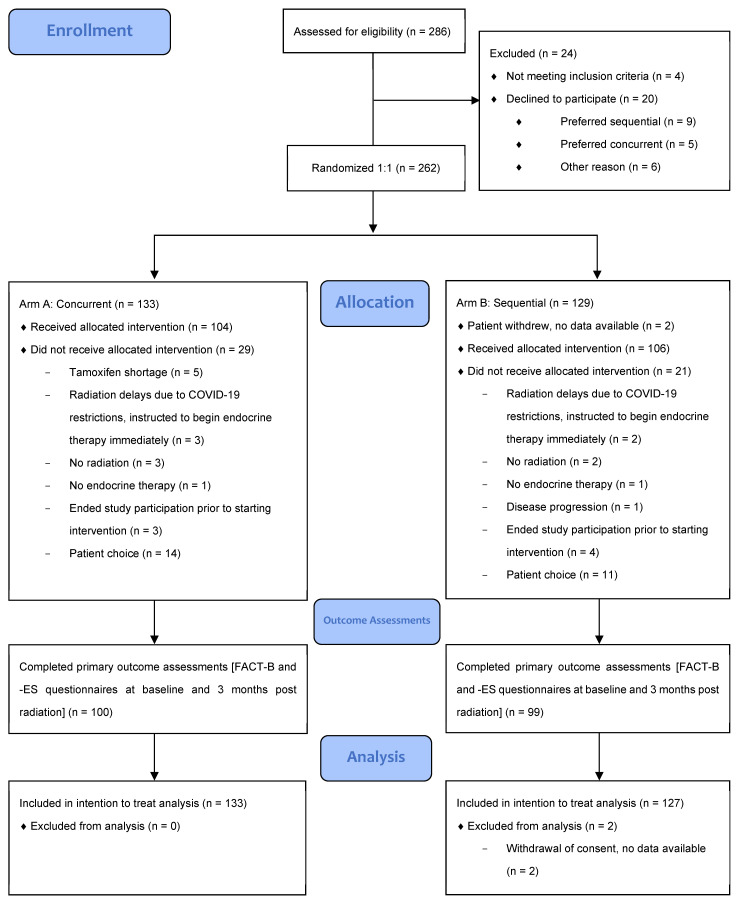
CONSORT 2010 flow diagram.

From September 2019 to January 2021, 133 patients were randomized to concurrent endocrine and radiotherapy, and 129 to sequential treatment with radiotherapy followed by endocrine therapy. The last participant completed follow-up on 6 April 2022. Two patients allocated to the sequential treatment arm were excluded from analysis as they withdrew immediately after enrollment and did not consent to study follow-up or data collection. As such, all analyses were based on the n = 127 patients in the sequential arm with available data. The CONSORT flow diagram is shown in Figure 1.

### 3.2. Baseline Characteristics

#### 3.2.1. Patient and Disease Characteristics

Baseline characteristics are presented in Table 1. The mean age of participants was 60 years, and all were female except one male in the sequential arm, with most patients being post-menopausal at enrollment (72.7%, 189/260). Sixty-six percent (171/260) had clinical stage I disease, with the majority being grade 2 (50.8%, 132/260). All patients were estrogen receptor (ER)-positive, with 12.3% (32/260) also being positive for human epidermal growth factor receptor 2 (HER2). Baseline QOL was high for most patients across FACT-ES, TOI, and EQ-5D-5L scores (Supplemental Materials Table S1).

#### 3.2.2. Treatment Characteristics

Treatment of the primary tumor consisted of lumpectomy for 81.5% of patients (212/260). Of these, one patient later additionally underwent mastectomy. In total, 18.5% (48/260) of patients underwent mastectomy. One patient in the sequential treatment arm was enrolled with a second breast primary that was treated with chest wall resection following prior mastectomy and reconstruction. Eighty-nine percent (232/260) had a sentinel lymph node biopsy (SLNB). Of these, 11 patients later underwent an axillary dissection. In total, 14.2% (37/260) of participants had an axillary dissection. Two participants did not undergo nodal surgery. Chemotherapy was received by 37.3% of patients (97/260), and 11% (28/260) received HER2-targeted therapy with trastuzumab.

Tamoxifen was the endocrine therapy of choice for 69.6% of patients (181/260), with an AI in the remainder (29.2%, 76/260), either letrozole or anastrozole. Two participants declined endocrine therapy, and one was found to have metastatic disease prior to starting treatment and therefore withdrew from the trial. Ovarian suppression in addition to endocrine therapy was received by 3% of patients (8/260). Seventy-five percent of patients received breast radiation (195/260), with the remainder receiving chest wall radiation (18.1%, 47/260). The median total radiation dose and fractions received were 40.1 Gray (range 26–50) and 15 fractions (range 5–25), respectively, although most, 206/242 (85.1%) received 40 Gy over 15 days. Axillary radiation was received by 32.7% (85/260), with 24.6% (64/260) receiving a boost to the tumor bed. Thirteen participants withdrew prior to receiving radiation (patient choice [12] and disease progression [1]), so the type of radiation was unknown. Five participants that were randomized to the study did not receive radiotherapy after being enrolled and withdrew (physician choice [4] and patient choice [1]).

### 3.3. Trial Compliance and Questionnaire Completion

The response rates for trial questionnaires and assessments at all study timepoints are reported in the Supplemental Materials (Supplemental Materials Table S2). The total completion rates for endocrine toxicity and QOL questionnaires (FACT-B/ES and EQ-5D-5L) at 3 months post radiotherapy were ≥79% (206/260) and were similarly high at other timepoints. Completion of radiation toxicity and cosmesis assessments, which required a physical breast exam, was negatively impacted by the transition to virtual care mandated by public health during the COVID-19 pandemic, with <50% of assessments available at the end of radiation and 3 and 6 months post radiation.

### 3.4. Primary Outcome

#### Change in Total FACT-ES Scores at 3 Months

For the primary outcome of change in endocrine therapy toxicity from baseline to 3 months post radiotherapy per total FACT-ES scores, there were 100 concurrent and 99 sequential patients with available data. No significant difference was found between patients treated with concurrent versus sequential endocrine and radiotherapy (median [range] = −4.9 (−82, 38.8) for concurrent and −5.1 (−42, 40) for sequential; *p* = 0.52 using ANCOVA, *p* = 0.87 using Wilcoxon rank sum test) (Table 2). Supportive analyses based on imputing results for missing data gave similar results. 

### 3.5. Secondary Outcomes

#### 3.5.1. Additional QOL Assessments

No significant difference was found between the groups in terms of FACT-TOI scores (*p*-value = 0.37 using ANCOVA, see Table 2 for other analyses) or any of the EQ-5D-5L domains from baseline to 3 months post radiotherapy (Table 3). Analysis also looked at the changes in total FACT-ES and TOI scores from baseline to other study timepoints (end of radiation, 6, and 12 months post radiation). The only statistically significant difference noted was in total FACT-ES scores at the end of radiation, with a greater decrease in QOL in the concurrent arm (median [range] = −5 (−55, 28) for concurrent and −2.3 (−40, 23) for sequential; *p* = 0.043) (Table 2). There were no significant differences in any EQ-5D-5L domains over time (Supplemental Materials Table S3).

#### 3.5.2. Radiation Toxicity

The transition to virtual care necessitated by the COVID-19 pandemic resulted in a significant lack of radiation toxicity and cosmesis assessments, making it difficult to accurately compare radiation toxicity between the groups. The most data available were at the end of study, 12 months post radiation, where 59% of assessments were completed. Data at this time were similar between the concurrent and sequential treatment groups, for all radiation toxicities assessed including rash, induration, pain, telangiectasia, swelling, fat necrosis, chronic mastitis, dyspnea, pneumonitis, and breast cosmesis (Supplemental Materials Table S4).

#### 3.5.3. Patient Experience and Preference

The response rate for the end of study patient questionnaire was high, at 75.2% (100/133) in the concurrent group and 78% (99/127) in the sequential group, with results provided in Table 4. Ninety-nine percent (257/260) of patients had started endocrine therapy, and the majority remained on treatment at the end of the study (90% [90/100] concurrent and 88.5% [85/96] sequential). Most patients did not think that the timing of radiation and endocrine therapy affected their ability to receive endocrine therapy (79% [79/100] concurrent and 67.4% [64/95] sequential) and had no preference for when adjuvant endocrine therapy should be started relative to radiotherapy (57.9% [55/95] concurrent and 65.3% [62/95] sequential).

## 4. Discussion

Although adjuvant radiation and endocrine therapy are established treatments in EBC, and guidelines suggest they may be delivered safely together [2], concerns remain about the optimal sequence of therapies. One concern regarding concurrent radiotherapy and endocrine therapy is the potential for increased toxicity with radiation. In a systematic review, we found no significant evidence of increased treatment-related toxicity with concurrent aromatase inhibitors or tamoxifen and radiotherapy [1]. Previous studies have suggested an increased risk of breast and lung fibrosis with concurrent radiation and tamoxifen, as tamoxifen may potentiate normal, post-radiation tissue retraction and fibrosis through activation of TGF-beta signaling [22,23]. However, the data are overall of poor quality, including retrospective studies using dated radiation techniques with many comparing concurrent treatment with radiation alone [1]. The argument is that if given sequentially, tamoxifen following radiation may still potentiate normal, post-radiation tissue fibrosis, yielding an equivalent risk of fibrosis regardless of the sequence. 

Upon surveying physicians, we found that another significant concern regarding concurrent radiation and endocrine therapy was the potential for increased endocrine therapy toxicities to interfere with treatment, which has been noted elsewhere in the literature [5,6]. Although a mechanism for this has not been postulated, concurrent treatment may lead to more fatigue and stress, which can exacerbate vasomotor symptoms and other endocrine therapy toxicities [7,8]. As endocrine therapy toxicity is an important clinical question not yet addressed in the literature on adjuvant radiation and endocrine therapy sequencing to date, this was selected as the primary endpoint of our trial. Furthermore, at the time of design, two randomized trials were in progress investigating concurrent versus sequential radiation and endocrine therapy with tamoxifen (CONSET, NCT00896155) and arimidex (STARS, NCT00887380), with endpoints including treatment efficacy (e.g., locoregional and distant failure) and toxicity including lung fibrosis and cosmesis. Of note, data from these studies have not yet been published, and the former has not been updated since 2011; thus, its status is unknown.

Endocrine therapy toxicity and breast cancer-specific QOL were assessed using the total FACT-ES and TOI scores, as validated in the seminal publications on endocrine therapy toxicity and QOL from the Arimidex, Tamoxifen, Alone or in Combination (ATAC) adjuvant breast cancer trial [18,19]. Data showed that for patients commencing endocrine therapy concurrently with, or sequential to, radiotherapy, there was no difference in endocrine therapy toxicity per total FACT-ES score, or overall breast cancer-specific QOL per TOI, from baseline to 3 months post radiotherapy. The only statistically significant difference noted between the groups over time was in the total FACT-ES scores at the end of radiation, which favored sequential treatment. However, many patients in the sequential group may have just started endocrine therapy, if at all; thus, it would be expected that endocrine toxicities would be more prevalent in the concurrent treatment group where all patients had started endocrine therapy.

Comparison of radiation toxicity was a secondary endpoint that was significantly limited by the number of toxicity assessments completed following the transition to virtual care mandated by public health during the COVID-19 pandemic, which did not allow for physical breast examination. Data at the end of study, where 59% of assessments were complete, suggested no significant difference in radiation toxicity between the concurrent and sequential treatment groups. Due to missing data, it was not possible to accurately assess differences in radiation toxicity at other timepoints. However, a previous randomized study showed no increase in radiation toxicity with concurrent versus sequential adjuvant radiation and endocrine therapy as the primary endpoint, with a median follow-up of 74 months [2,3]. Furthermore, it is hoped that the above active trials will further address this important issue. 

The rates of patients remaining on endocrine therapy at the end of study (12 months post radiation) were high, at over 88%, and similar between the treatment groups. Furthermore, self-reported compliance with endocrine therapy was high, with most patients stating they never, or rarely, missed a dose and had no difficulties taking the medication. This may reflect the clinical trial environment where patients had, or perceived, greater clinical contact and support from healthcare providers and research teams. However, the literature reports rates of discontinuation and non-compliance with endocrine therapy in clinical practice as high as 20 to 50% [24,25]. This lends further importance to identifying initiatives to support patients to remain on curative endocrine therapies, through efforts such as this study, to prevent and manage significant treatment-related toxicities. Importantly, most patients did not think the timing of radiation and endocrine therapy impacted their ability to receive endocrine therapy and had no preference for how therapies should be sequenced.

This study does have important limitations, the most significant being the lack of sufficient data on radiation toxicity due to the transition to virtual care during the COVID-19 pandemic. Alternate means to assess these endpoints remotely could have been implemented e.g., inclusion of skin and lung symptom assessment and QOL scales. However, given that the COVID-19 pandemic began 6 months into recruitment with 122 patients enrolled (56% of the target sample size of 218), we did not feel that an amendment to the study protocol to include such measures would have had a meaningful impact on results. Conducting a trial throughout the COVID-19 pandemic did, however, provide our group with invaluable lessons for incorporating virtual care into clinical trials and research going forward [26].

An additional limitation is that most patients received tamoxifen therapy, limiting our assessment of concurrent radiation and aromatase inhibition. This was particularly the case with respect to endocrine therapy toxicities, which have not previously been reported in the literature. However, to ensure the trial remained pragmatic, we did not dictate the choice of endocrine therapy. Data have shown similar survival outcomes with 5 years of an aromatase inhibitor (“upfront” strategy) compared with treatment incorporating both tamoxifen and an aromatase inhibitor (“switch” strategy), but with a reduced risk of osteoporosis, fracture, and other side effects [27,28]. The “switch” strategy has consequently become a common, guideline-endorsed [29], clinical practice and this probably explains the increased use of tamoxifen seen in our study. In this pragmatic study, there were variations in the total doses, fraction size, and number of fractions received. This reflects the trial design, which did not mandate the nature of the locoregional radiotherapy prescribed. It also reflects the unique time in clinical practice that the trial was conducted, with the emergence of hypofractionation (e.g., higher doses per fraction) in adjuvant breast radiation coinciding with the COVID-19 pandemic. Thus, in response to public health mandates to reduce healthcare contact, and to patients’ preferences, shorter courses of adjuvant breast radiation were often prescribed. This was in keeping with clinical practice guidelines at the time, which stated that hypofractionated regimens should be strongly considered whenever possible [30]. Most patients in this study received standard whole breast radiation, with 40 Gy delivered over 15 days. As the use of hypofractionated radiation treatment regimens (e.g., higher doses per fraction) becomes more widespread, it will be important to have greater data on the optimal sequence of therapies in this situation. A limitation of the analysis was that we did not stratify by the specific treatment received (e.g., type of surgery, radiation, or endocrine therapy). However, given that most patients were prescribed tamoxifen and received similar radiation for stage I disease, we do not think this would have importantly impacted the findings.

Finally, two patients in the sequential arm withdrew immediately following randomization and did not allow any data collection, while substantial numbers of patients dropped out over time. This resulted in additional amounts of missing data for all outcomes.

## 5. Conclusions

To our knowledge, this is the first clinical trial to investigate the impact of concurrent versus sequential radiation and endocrine therapy in EBC on endocrine therapy toxicity, and no significant difference was found. This adds further data to support the safety and tolerability of adjuvant radiation and endocrine therapy regardless of the sequence of therapy, allowing patients and physicians to determine the optimal timing of therapies for the individual patient, as highlighted in 2024 ESMO clinical practice guidelines [29].

## Figures and Tables

**Table 1:** Baseline characteristics.

		No.	All Patients (n: 260)	Concurrent (n: 133)	Sequential (n: 127)
**Patient Characteristics**					
Age	Mean (SD)	260	60.4 (11.0)	59.6 (11.6)	61.2 (10.4)
Sex	No. (%) Female	260	259 (99.6)	133 (100.0)	126 (99.2)
Menopausal Status	Male		1 (0.4)	0	1 (0.8)
	Peri-	260	23 (8.9)	13 (9.8)	10 (7.9)
	Post-		189 (72.7)	93 (69.9)	96 (75.6)
	Pre-		40 (15.4)	23 (17.3)	17 (13.4)
	Unknown		8 (3.1)	4 (3.0)	4 (3.2)
**Disease Characteristics**					
Tumor Size (mm)	Median (range)	260	18 (2, 104)	18 (4, 100)	18 (2, 104)
T Stage (Clinical)	T1	260	157 (60.4)	81 (60.9)	76 (59.8)
	T2		79 (30.4)	39 (29.3)	40 (31.5)
	T3		18 (6.9)	10 (7.5)	8 (6.3)
	T4		6 (2.3)	3 (2.3)	3 (2.4)
N Stage (Clinical)	N0	260	166 (63.9)	85 (63.9)	81 (63.8)
	N1		78 (30.0)	38 (28.6)	40 (31.5)
	N2		10 (3.9)	6 (4.5)	4 (3.2)
	N3		6 (2.3)	4 (3.0)	2 (1.6)
Overall Stage (Clinical)	IA	260	125 (48.1)	65 (48.9)	60 (47.2)
	IB		46 (17.7)	22 (16.5)	24 (18.9)
	IIA		50 (19.2)	21 (15.8)	29 (22.8)
	IIB		19 (7.3)	13 (9.8)	6 (4.7)
	IIIA		12 (4.6)	7 (5.3)	5 (3.9)
	IIIB		7 (2.7)	4 (3.0)	3 (2.4)
	IIIC		1 (0.4)	1 (0.8)	0
Grade	1	260	60 (23.1)	28 (21.1)	32 (25.2)
	2		132 (50.8)	63 (47.4)	69 (54.3)
	3		68 (26.2)	42 (31.6)	26 (20.5)
Breast Markers	ER Positive	260	260 (100.0)	133 (100.0)	127 (100.0)
	PR Positive		222 (85.4)	116 (87.2)	106 (83.5)
	HER2 Positive		32 (12.3)	13 (9.8)	19 (15.0)
**Treatment Characteristics**					
Surgery	Mastectomy	260	48 (18.5)	23 (17.3)	25 (19.7)
	Lumpectomy		212 (81.5)	110 (82.7)	103 (81.1)
	Chest Wall Resection		1 (0.4)	0	1 (0.84)
	Breast Reconstruction		28 (10.8)	9 (6.8)	19 (15.0)
	SLNB		232 (89.2)	118 (88.7)	114 (89.8)
	Axillary Dissection		37 (14.2)	21 (15.8)	16 (12.6)
	No nodal surgery		2 (0.8)	0	2 (1.6)
Chemotherapy	Adjuvant	260	66 (25.4)	39 (29.3)	27 (21.3)
	Neoadjuvant		31 (11.9)	13 (9.8)	18 (14.2)
HER2 Therapy	Trastuzumab	260	28 (10.8)	12 (9.0)	16 (12.6)
Endocrine Therapy	Anastrozole	260	41 (15.8)	23 (17.3)	18 (14.2)
	Letrozole		35 (13.5)	13 (9.8)	22 (17.3)
	Tamoxifen		181 (69.6)	96 (72.2)	85 (66.9)
	Not Received		3 (1.2)	1 (0.08)	2 (1.6)
Ovarian Suppression	Yes	260	8 (3.1)	2 (1.5)	6 (4.7)
Radiation	Breast	260	195 (75)	102 (76.7)	93 (73.2)
	Chest Wall		47 (18.1)	23 (17.3)	24 (18.9)
	Lymph Nodes		85 (32.7)	43 (32.3)	42 (33.1)
	Boost		64 (24.6)	39 (29.3)	25 (19.7)
	Not Received		5 (1.9)	3 (2.3)	2 (1.6)
	Unknown		13 (5)	5 (3.8)	8 (6.3)
Breast/Chest Wall Total Dose	Median (range)	242	40.1 (26, 50)	40.1 (26.0, 50.0)	40.1 (26, 50)
Breast/Chest Wall No. Fractions	Median (range)	242	15 (5, 25)	15 (5, 25)	15 (5, 25)
Lymph Nodes Total Dose	Median (range)	85	40.1 (26.5, 50.0)	40.1 (26.5, 50.0)	40.1 (40.1, 50)
Lymph Nodes No. of Fractions	Median (range)	85	15 (5, 25)	15 (5, 25)	15 (15, 25)
Boost Total Dose	Median (range) ^1^	64	10 (10, 48)	10 (10, 48)	10 (10, 48)
Boost No. of Fractions	Median (range) ^1^	64	4 (4, 15)	4 (4, 15)	5 (4, 15)

Abbreviations: estrogen receptor (ER); human epidermal growth factor receptor 2 (HER2); progesterone receptor (PR); standard deviation (SD); sentinel lymph node biopsy (SLNB). ^1^ Data on total dose and fractions include patients receiving concomitant and sequential boost; thus, higher ranges are reported.

**Table 2:** Median change (range) in FACT QOL scores over time.

	Total FACT-ES			FACT-TOI		
	Concurrent	Sequential	*p* Value	Concurrent	Sequential	*p* Value
3 months post RT	−4.9 (−82, 38.8)	−5.1 (−42, 40)	0.87	0.2 (−40, 33)	−1.4 (−21, 24)	0.18
End RT	**−5** (**−55**, **28**)	**−2.3** (**−40**, **23**)	**0.043**	−2 (−35, 22)	−2 (−28, 18)	0.76
6 months post RT	−8 (−79, 37.8)	−4 (−45, 38)	0.50	−1 (−39, 38)	−3 (−30, 31)	0.36
12 months post RT	−6.3 (−87, 35.2)	−6.4 (−57.9, 43)	0.68	0.4 (−44, 35)	−0.2 (−23, 42)	0.54

FACT *p*-values are from Wilcoxon rank sum test using the change from baseline. Statistically significant findings in bold. Abbreviations: endocrine symptoms (ES). Functional Assessment of Cancer Therapy (FACT); radiotherapy (RT); trial outcome index (TOI).

**Table 3:** Change in EQ-ED-5L responses from baseline to 3 months post radiotherapy.

		No (%)	Baseline		No (%)	3 Months Post RT		*p* Value
			Concurrent	Sequential		Concurrent	Sequential	
EQ-5D-5L Mobility	No problems	223	87 (78.4)	87 (77.7)	205	84 (79.3)	75 (75.8)	0.52
	Slight		17 (15.3)	13 (11.6)		15 (14.2)	15 (15.2)	
	Moderate		5 (4.5)	11 (9.8)		5 (4.7)	7 (7.1)	
	Severe		2 (1.8)	1 (0.9)		2 (1.9)	2 (2.0)	
	Unable		0	0		0	0	
EQ-5D-5L Selfcare	No problems	223	97 (87.4)	107 (95.5)	205	96 (91.4)	94 (94.0)	0.39
	Slight		12 (10.8)	2 (1.8)		3 (2.9)	3 (3.0)	
	Moderate		2 (1.8)	2 (1.8)		6 (5.7)	3 (3.0)	
	Severe		0	1 (0.9)		0	0	
	Unable		0	0		0	0	
EQ-5D-5L Usual Activities	No problems	223	67 (60.4)	67 (59.8)	206	69 (65.1)	60 (60.0)	0.41
	Slight		29 (26.1)	35 (31.3)		26 (24.5)	29 (29.0)	
	Moderate		13 (11.7)	9 (8.0)		9 (8.5)	6 (6.0)	
	Severe		2 (1.8)	1 (0.9)		2 (1.9)	5 (5.0)	
	Unable		0	0		0	0	
EQ-5D-5L Pain/Discomfort	No problems	223	42 (37.8)	40 (35.7)	206	29 (27.4)	24 (24.0)	0.60
	Slight		46 (41.4)	57 (50.9)		51 (48.1)	47 (47.0) 23 (23.0)	
	Moderate		21 (18.9)	10 (8.9)		18 (17.0)	5 (5.0)	
	Severe		2 (1.8)	3 (2.7)		7 (6.6)	1 (1.0)	
	Extreme		0	2 (1.8)		1 (0.9)		
EQ-5D-5L Anxiety/Depression	No problems	221	47 (43.1)	50 (44.6)	204	45 (42.9)	52 (52.5)	0.16
	Slight		45 (41.3)	49 (43.8)		41 (39.1)	33 (33.3)	
	Moderate		13 (11.9)	12 (10.7)		16 (15.2)	13 (13.10)1 (1.0)	
	Severe		4 (3.7)	0		3 (2.9)	0	
	Extreme		0	1 (0.9)		0		
EQ-5D-5L Health Today ^1^	0	217	0	1 (0.9)	202	0	0	0.67
	1–49		5 (4.7)	4 (3.6)		7 (6.8)	4 (4.0)	
	50–79		47 (43.9)	41 (37.3)		34 (33.0)	41 (41.4)	
	80–99		50 (46.7)	63 (57.3)		59 (57.3)	52 (52.5)	
	100		5 (4.7)	1 (0.9)		3 (2.9)	2 (2.0)	

EQ-5D-5L *p*-values are from the Cochran–Armitage test for trend. ^1^ Health today rated on a scale from 0–100, where 100 is the best imaginable health.

**Table 4:** End-of-study patient questionnaire.

Questions	Answers	Responses No. (%)	
		Concurrent (n: 100)	Sequential (n: 99)
*Have you started taking the prescribed ET?*	**Yes**	**100** (**100**)	**99** (**100**)
*Are you still taking the prescribed ET?*	**Yes**	**90** (**90.0**)	**85** (**88.5**)
	No	10 (10.0)	11 (11.5)
	N/A	0	0
*How often would you miss an ET dose?*	**Never**	**67** (**71.3**)	**70** (**74.5**)
	Once/month	14 (14.9)	13 (13.8)
	Once/week	1 (1.1)	3 (3.2)
	Several times a week	0	2 (2.1)
	I don’t know	6 (6.4)	1 (1.1)
	N/A	6 (6.4)	5 (5.3)
*Missing a few doses is not a big deal?*	Agree	15 (15.6)	11 (11.5)
	**Disagree**	**47** (**49.0**)	**45** (**46.9**)
	I don’t know	34 (35.4)	40 (41.7)
*Have you found it difficult to take the prescribed ET?*	Yes	24 (24.7)	22 (22.7)
	**No**	**69** (**71.1**)	**74** (**76.3**)
	I don’t know	3 (3.1)	1 (1.0)
	N/A	1 (1.0)	0
*What makes it difficult to take ET?*	I don’t like taking medications	3	1
	I forget to take it	1	2
	Causes me bothersome symptoms	18	21
	Duration of treatment is too long	2	2
	Cost	0	0
	Other	7	2
	**No problems**	**23**	**27**
	N/A	10	11
*Has your doctor made changes to your ET?*	Time off treatment	17	15
	Reduction in dose	24	15
	Every other day dosing	1	6
	Change to a different ET	7	3
	I don’t know	0	0
	**N/A**	**63**	**70**
*Would anything make taking ET easier?*	More follow-up/clinical contact	12	9
	More help with side effects	19	24
	Shorter duration of treatment	13	12
	Greater information	25	15
	Other	2	6
	**I don’t know**	**31**	**28**
*Did the timing of RT affect your ability to take ET?*	Yes	0	1 (1.1)
	**No**	**79** (**79.0**)	**64** (**67.4**)
	I don’t know	21 (21.0)	30 (31.6)
*When do you think ET should start relative to RT?*	Before RT	27 (28.4)	1 (1.1)
	After RT	13 (13.7)	32 (33.7)
	**No Preference**	**55** (**57.9**)	**62** (**65.3**)

Abbreviations: endocrine therapy (ET); radiation therapy (RT). Majority responses in bold.

## Data Availability

The de-identified dataset is available upon request and approval by the Ontario Cancer Research Ethics Board (OCREB 1-866-678-6427 Ext 6649).

## Supplementary Materials

The following supporting information can be downloaded at https://www.mdpi.com/article/10.3390/curroncol31080338/s1, Table S1: Baseline EQ-5D-5L and FACT scores; Table S2: Trial compliance with questionnaires and assessments; Table S3: Changes in EQ-ED-5L responses over time; Table S4: Comparison of radiation toxicities at baseline and end of study.
