# Supplementary material for: A Randomized Trial Comparing Concurrent versus Sequential Radiation and Endocrine Therapy in Early-Stage, Hormone-Responsive Breast Cancer
Source: Curr Oncol. 2024 Aug 7;31(8):4531–45. doi: 10.3390/curroncol31080338 (PMC11353105; doi:10.3390/curroncol31080338)
Supplement: Supplementary file 1 [file curroncol-31-00338-s001.zip › curroncol-3076538-supplementary.pdf]

SUPPLEMENTARY MATERIALS

Table S1. Baseline EQ-5D-5L and FACT scores.

QOL Score	Rating	No.	All Patients	Concurrent	Sequential
**Total FACT-ES**<br>*(Score Range: 0-184)*	Median (range)	215	151 (70, 183)	149.3 (70, 181)	153 (82, 183)
**FACT-TOI**<br>*(Score Range: 0-96)*	Median (range)	217	71 (28, 95)	70 (29, 95)	72.5 (28, 94.6)
**EQ-5D-5L**<br>**Mobility**	No problems<br>Slight<br>Moderate<br>Severe<br>Unable To Do	223	174 (78.0)<br>30 (13.5)<br>16 (7.2)<br>3 (1.4)<br>0	87 (78.4)<br>17 (15.3)<br>5 (4.5)<br>2 (1.8)<br>0	87 (77.7)<br>13 (11.6)<br>11 (9.8)<br>1 (0.9)<br>0
**EQ-5D-5L**<br>**Selfcare**	No problems<br>Slight<br>Moderate<br>Severe<br>Unable To Do	223	204 (91.5)<br>14 (6.3)<br>4 (1.8)<br>1 (0.5)<br>0	97 (87.4)<br>12 (10.8)<br>2 (1.8)<br>0<br>0	107 (95.5)<br>2 (1.8)<br>2 (1.8)<br>1 (0.9)<br>0
**EQ-5D-5L**<br>**Usual Activities**	No problems<br>Slight<br>Moderate<br>Severe<br>Unable To Do	223	134 (60.1)<br>64 (28.7)<br>22 (9.9)<br>3 (1.4)<br>0	67 (60.4)<br>29 (26.1)<br>13 (11.7)<br>2 (1.8)<br>0	67 (59.8)<br>35 (31.3)<br>9 (8.0)<br>1 (0.9)<br>0
**EQ-5D-5L**<br>**Pain/Discomfort**	No problems<br>Slight<br>Moderate<br>Severe<br>Extreme	223	82 (36.8)<br>103 (46.2)<br>31 (13.9)<br>5 (2.2)<br>2 (0.9)	42 (37.8)<br>46 (41.4)<br>21 (18.9)<br>2 (1.8)<br>0	40 (35.7)<br>57 (50.9)<br>10 (8.9)<br>3 (2.7)<br>2 (1.8)
**EQ-5D-5L**<br>**Anxiety/Depression**	No problems<br>Slight<br>Moderate<br>Severe<br>Extreme	221	97 (43.9)<br>94 (42.5)<br>25 (11.3)<br>4 (1.8)<br>1 (0.5)	47 (43.1)<br>45 (41.3)<br>13 (11.9)<br>4 (3.7)<br>0	50 (44.6)<br>49 (43.8)<br>12 (10.7)<br>0<br>1 (0.9)
**EQ-5D-5L**<br>**Health Today***	0<br>1-49<br>50-79<br>80-99<br>100	217	1 (0.5)<br>9 (4.2)<br>88 (40.5)<br>113 (41.1)<br>6 (2.8)	0<br>5 (4.7)<br>47 (43.9)<br>50 (46.7)<br>5 (4.7)	1 (0.9)<br>4 (3.6)<br>41 (37.3)<br>63 (57.3)<br>1 (0.9)

<u>*Abbreviations*</u>: Endocrine Symptoms (ES). Functional Assessment of Cancer Therapy (FACT). Quality of Life (QOL). Trial Outcome Index (TOI). *Health today rated on scale from 0-100, where 100 is best imaginable health.

Table S2. Trial compliance with questionnaires and assessments.

	No.	Baseline<br>No. (%)	End Radiation<br>No. (%)	3mos post Radiation<br>No. (%)	6mos post Radiation<br>No. (%)	12mos post Radiation<br>No. (%)
**TOTAL**	**260**					
FACT-B		221 (85.0)	222 (85.4)	208 (80.0)	213 (81.9)	199 (76.5)
FACT-ES		221 (85.0)	222 (85.4)	208 (80.0)	213 (81.9)	199 (76.5)
EQ-5D-5L		223 (85.8)	219 (84.2)	206 (79.2)	216 (83.1)	201 (77.3)
Patient Questionnaire		224 (86.2)	----	----	----	199 (76.5)
Physician Questionnaire		----	200 (76.9)	225 (86.5)	232 (89.2)	231 (88.8)
Radiation Toxicity		207 (79.6)	105 (40.4)	103 (39.6)	119 (45.8)	154 (59.2)
**CONCURRENT**	**133**					
FACT-B		109 (82.0)	112 (84.2)	106 (79.7)	106 (79.7)	100 (75.2)
FACT-ES		109 (82.0)	112 (84.2)	106 (79.7)	106 (79.7)	100 (75.2)
EQ-5D-5L		111 (83.5)	110 (82.7)	106 (79.7)	108 (81.2)	101 (75.9)
Patient Questionnaire		112 (84.2)	----	----	----	100 (75.2)
Physician Questionnaire		N/A	104 (78.2)	113 (85.0)	122 (91.8)	119 (89.5)
Radiation Toxicity		109 (82.0)	52 (39.1)	43 (32.3)	63 (47.4)	82 (61.7)
**SEQUENTIAL**	**127**					
FACT-B		112 (88.2)	110 (86.6)	102 (80.3)	107 (84.2)	99 (78.0)
FACT-ES		112 (88.2)	110 (86.6)	102 (80.3)	107 (84.2)	99 (78.0)
EQ-5D-5L		112 (88.2)	109 (85.8)	100 (78.7)	108 (85.0)	100 (78.7)
Patient Questionnaire		112 (88.2)	----	----	----	99 (78.0)
Physician Questionnaire		----	96 (75.6)	112 (88.2)	110 (86.6)	112 (88.2)
Radiation Toxicity		98 (77.2)	53 (41.7)	60 (47.2)	56 (44.1)	72 (56.7)

Abbreviations: Breast (B). Endocrine Symptoms (ES). Functional Assessment of Cancer Therapy (FACT).

Table S3. Change in EQ-ED-5L responses over time.

		End RT			6 months Post RT			12 months post RT		
		**Con**	**Seq**	**P value**	**Con**	**Seq**	**P value**	**Con**	**Seq**	**P value**
**EQ-5D-5L Mobility**	No problems<br>Slight<br>Moderate<br>Severe<br>Unable To Do	84 (76.4)<br>15 (13.6)<br>9 (8.2)<br>2 (1.8)<br>0	80 (73.4)<br>20 (18.4)<br>6 (5.5)<br>3 (2.8)<br>0	0.82	79 (73.2)<br>16 (14.8)<br>10 (9.3)<br>3 (2.8)<br>0	75 (69.4)<br>20 (18.5)<br>10 (9.3)<br>3 (2.8)<br>0	0.72	78 (77.2)<br>15 (14.9)<br>6 (5.9)<br>2 (2.0)<br>0	69 (69.0)<br>21 (21.0)<br>8 (8.0)<br>2 (2.0)<br>0	0.30
**EQ-5D-5L Selfcare**	No problems<br>Slight<br>Moderate<br>Severe<br>Unable To Do	99 (90.0)<br>10 (9.1)<br>1 (0.9)<br>0<br>0	101 (92.7)<br>4 (3.7)<br>4 (3.7)<br>0<br>0	0.98	98 (90.7)<br>7 (6.5)<br>2 (1.9)<br>1 (0.9)<br>0	99 (91.7)<br>5 (4.6)<br>3 (2.8)<br>1 (0.9)<br>0	1.00	88 (87.1)<br>9 (8.9)<br>3 (3.0)<br>1 (1.0)<br>0	91 (91.0)<br>6 (6.0)<br>3 (3.0)<br>0<br>0	0.38
**EQ-5D-5L Usual Activities**	No problems<br>Slight<br>Moderate<br>Severe<br>Unable To Do	56 (50.9)<br>35 (31.8)<br>18 (16.4)<br>1 (0.9)<br>0	62 (56.9)<br>30 (27.5)<br>15 (13.8)<br>2 (1.8)<br>0	0.53	67 (62.0)<br>26 (24.1)<br>14 (13.0)<br>1 (0.9)<br>0	70 (64.8)<br>28 (25.9)<br>7 (6.5)<br>3 (2.8)<br>0	0.58	68 (67.3)<br>24 (23.8)<br>7 (6.9)<br>2 (2.0)<br>0	67 (67.0)<br>22 (22.0)<br>9 (9.0)<br>2 (2.0)<br>0	0.81
**EQ-5D-5L Pain/ Discomfort**	No problems<br>Slight<br>Moderate<br>Severe<br>Extreme	24 (21.8)<br>50 (45.5)<br>29 (26.4)<br>7 (6.4)<br>0	28 (25.7)<br>46 (42.2)<br>28 (25.7)<br>6 (5.5)<br>1 (0.9)	0.76	33 (30.6)<br>49 (45.4)<br>18 (16.7)<br>8 (7.4)<br>0	24 (22.2)<br>55 (50.9)<br>24 (22.2)<br>4 (3.7)<br>1 (0.9)	0.42	31 (30.7)<br>46 (45.5)<br>19 (18.8)<br>4 (4.0)<br>1 (1.0)	34 (34.0)<br>39 (39.0)<br>22 (22.0)<br>5 (5.0)<br>0	0.93
**EQ-5D-5L Anxiety/ Depression**	No problems<br>Slight<br>Moderate<br>Severe<br>Extreme	46 (41.8)<br>50 (45.5)<br>12 (10.9)<br>2 (1.8)<br>0	51 (46.8)<br>43 (39.5)<br>14 (12.8)<br>0<br>1 (0.9)	0.70	47 (43.5)<br>39 (36.1)<br>18 (16.7)<br>4 (3.7)<br>0	52 (48.2)<br>42 (38.9)<br>13 (12.0)<br>1 (0.9)<br>0	0.17	43 (42.6)<br>36 (35.6)<br>19 (18.8)<br>3 (3.0)<br>0	49 (49.0)<br>41 (41.0)<br>8 (8.0)<br>2 (2.0)<br>0	0.083

**EQ-5D-5L Health Today***	0<br>1-49<br>50-79<br>80-99<br>100	0<br>4 (3.6)<br>55 (50.0)<br>47 (42.7)<br>4 (3.6)	0<br>2 (1.8)<br>49 (45.0)<br>55 (50.5)<br>3 (2.8)	0.34	0<br>7 (6.6)<br>34 (32.1)<br>62 (58.5)<br>3 (2.8)	0<br>3 (2.9)<br>38 (36.5)<br>62 (59.6)<br>1 (1.0)	0.90	0<br>4 (4.0)<br>35 (34.7)<br>59 (58.4)<br>3 (3.0)	0<br>2 (2.1)<br>35 (36.1)<br>58 (59.8)<br>2 (2.1)	0.86

EQ-5D-5L p-values are from Cochran-Armitage test for trend. Each time point compared with baseline. *Health today rated on scale from 0-100, where 100 is best imaginable health.

Table S4. Comparison of radiation toxicities at baseline and end of study.

		Baseline (n:207)		12 Months Post RT (n:154)		
**Toxicity**	**Grade**	**Con. No. (%)**	**Seq. No. (%)**	**Con. No. (%)**	**Seq. No. (%)**	**P value**
**Rash**	**1**<br>**2**<br>**3**	5 (4.6)<br>0<br>0	4 (4.1)<br>0<br>0	10 (12.2)<br>0<br>0	10 (14.1)<br>0<br>0	0.81
**Induration**	**1**<br>**2**<br>**3**	27 (25.0)<br>5 (4.6)<br>0	24 (24.5)<br>6 (6.1)	23 (28.1)<br>5 (6.1)<br>1 (1.2)	22 (30.6)<br>7 (9.7)<br>0	0.62
**Pain**	**1**<br>**2**<br>**3**	25 (23.2)<br>1 (0.9)<br>0	20 (20.6)<br>3 (3.1)<br>0	22 (26.8)<br>2 (2.4)<br>2 (2.4)	17 (23.6)<br>0<br>0	0.29
**Telangiectasia**	**1**<br>**2**<br>**3**	1 (0.9)<br>0<br>0	0<br>0<br>0	3 (3.7)<br>1 (1.2)<br>0	2 (2.8)<br>0<br>0	0.69
**Breast Swelling**	**1**<br>**2**<br>**3**	25 (23.4)<br>1 (0.9)<br>0	20 (20.6)<br>0<br>0	16 (19.5)<br>5 (6.1)<br>0	13 (18.3)<br>2 (2.8)<br>0	0.57
**Fat Necrosis**	**1**<br>**2**<br>**3**	5 (4.6)<br>3 (2.8)<br>0	6 (6.1)<br>3 (3.1)<br>0	6 (7.3)<br>1 (1.2)<br>0	6 (8.5)<br>3 (4.2)<br>0	0.44
**Chronic Mastitis**	**1**<br>**2**<br>**3**	0<br>0<br>0	0<br>1 (1.0)<br>0	2 (2.4)<br>0<br>0	1 (1.4)<br>0<br>0	1.00
**Dyspnea**	**1**<br>**2**<br>**3**	7 (6.4)<br>0<br>0	4 (4.2)<br>1 (1.0)<br>0	1 (1.2)<br>0<br>0	2 (2.8)<br>0<br>0	0.60
**Pneumonitis**	**1**<br>**2**<br>**3**	0<br>0<br>0	0<br>0<br>0	0<br>0<br>0	0<br>0<br>0	NA
**Breast Cosmesis***	**1**<br>**2**<br>**3**	42 (40.8)<br>9 (8.7)<br>4 (3.9)	40 (46.0)<br>5 (5.8)<br>3 (3.5)	36 (48.0)<br>8 (10.7)<br>4 (5.3)	28 (42.4)<br>6 (9.1)<br>2 (3.0)	0.30

*Breast cosmesis per EORTC grading: (0) excellent (1) good (2) fair (3) poor. All other items graded per CTCAE: (1) mild (2) moderate (3) severe (4) life threatening or disabling (5) death related to adverse event. P-values are from Fisher's exact test, comparing any grade toxicity versus no toxicity between treatment groups at 12 months post radiation. *Abbreviations*: Radiation therapy (RT).
